# Precision prediction of hyperhomocysteinemia development in perimenopausal women using LASSO regression

**DOI:** 10.3389/frph.2025.1670141

**Published:** 2025-10-09

**Authors:** Xuan Tan, Mingqi Li, Jie Wang, Yiwei Peng, Liwen Zhu, Na Jiang, Ling Li, Xiuqin Hong

**Affiliations:** ^1^ Clinical Epidemiology Research Office, Hunan Provincial People’s Hospital (The First Affiliated Hospital of Hunan Normal University), Changsha, China; ^2^ Key Laboratory of Molecular Epidemiology, Hunan Normal University, Changsha, China

**Keywords:** hyperhomocysteinemia, LASSO, nomogram, perimenopausal women, factor associated

## Abstract

**Background:**

Hyperhomocysteinemia (HHcy) is associated with an increased risk of cardiovascular diseases, particularly in perimenopausal women, who are more susceptible to metabolic disorders due to declining estrogen levels. This study aimed to identify risk factors and develop a predictive model for HHcy in this population.

**Methods:**

A retrospective study included 687 perimenopausal women, divided into a training set (481) and an internal validation set (206). Demographic characteristics, pregnancy-related factors, lifestyles, and diet information were collected by questionnaire. 63 perimenopausal women hospitalized from March to June 2025 were selected as the external validation set. The least absolute shrinkage and selection operator (LASSO) regression was used to select variables. The logistic regression model was developed to predict HHcy risk, with results visualized using a nomogram. Model performance was evaluated using receiver operating characteristic (ROC) curves, calibration curves, and decision curve analysis (DCA).

**Results:**

137 of 687 (19.94%) perimenopausal women had HHcy. Through Lasso regression and multifactor logistic regression, 4 predictors were identified, including egg consumption frequency, LDL, TP, and CysC for constructing the nomogram model. The AUC of the training set was 0.765 (95% CI = 0.708–0.822), for the internal validation set was 0.854 (95% CI = 0.781–0.928), and for the external validation set was 0.776 (95% CI = 0.603–0.949), indicating good predictive performance of the model.

**Conclusion:**

The nomogram demonstrated high predictive accuracy and clinical utility, providing a potential tool for HHcy risk prediction and selection of treatment strategies in perimenopausal women.

## 1 Introduction

Perimenopause, or the menopausal transition, refers to the period during which physiological changes signal the progression toward a woman's final menstrual period. This phase begins with the onset of menstrual disorders and continues until a woman enters menopause, or one year after amenorrhea occurs (1). Women during this period are more susceptible to certain health risks due to declining estrogen levels, and one of the important health risks is hyperhomocysteinemia (HHcy) (2). As a blood biochemical predictor, elevated levels of homocysteine (Hcy) are closely associated with the risk of cardiovascular diseases and ischemic stroke (3, 4). Additionally, an evidence-based analysis confirmed that markedly elevated Hcy levels significantly increase the risk of developing type 2 diabetes (5). Studies have shown that postmenopausal women have an increased prevalence of HHcy (2)—a finding that aligns with the physiological changes characteristic of this stage and lays the groundwork for exploring HHcy risk in the perimenopausal transition.

Hcy levels are influenced by many factors, encompassing genetic predispositions, dietary intake, and lifestyle choices. Genetic factors are pivotal, with hereditary defects in metabolic and methylation pathways contributing to increased Hcy levels. Notably, the polymorphism of the methyltetrahydrofolate reductase (MTHFR) gene—specifically its 677TT genotype—is closely associated with Hcy levels (6). Dietary intake significantly influences Hcy levels. Vitamins B12, B6, and folic acid are essential coenzymes in Hcy metabolism, and their plasma levels are inversely correlated with plasma Hcy concentrations. Insufficient intake of these nutrients results in elevated plasma Hcy levels. Additionally, a diet rich in methionine, which is characteristic of high animal protein and low vegetable protein intake, may contribute to HHcy. Age, underlying diseases, and medication use also modulate Hcy levels, with levels generally increasing with age. Comorbidities such as hepatic or renal impairment, and the use of contraceptives, antiepileptic drugs, diuretics, and other medications can elevate Hcy levels (7). Additionally, our prior studies indicate that in clinical practice, estradiol (E2) is a protective factor of HHcy.

Perimenopausal women exhibit unique physiological and hormonal fluctuations, and while their risk of developing HHcy is elevated, it also shows inter-individual heterogeneity. These characteristics lead to greater variability in predictive factors among perimenopausal women; therefore, a robust variable selection method such as Least Absolute Shrinkage and Selection Operator (LASSO)-logistic regression is urgently needed to avoid model overfitting and ensure model stability. LASSO regression is a variable selection method proposed by statistician Robert Tibshirani in 1996 (8). Compared with traditional regression methods, LASSO regression can deal with a larger number of potential predictors and select the variables that are most relevant to the disease which is an important tool for clinical screening of influencing factors. Given the critical need for early identification of HHcy risk in perimenopausal women and the current lack of specialized predictive tools, the objective of our study was to explore associated risk factors using the LASSO regression method and to develop a predictive model. This predictive model was designed to integrate demographic characteristics, lifestyles, diets, pregnancy-related factors, and biochemical indicators to identify patients at high risk for developing HHcy before these perimenopausal women meet the full diagnostic criteria. The development of such a predictive model represents a crucial step toward improving the management of perimenopausal women who may develop HHcy, potentially reducing morbidity through early intervention.

## 2 Materials and methods

### 2.1 Study subjects

Perimenopausal women hospitalized at Hunan Provincial People's Hospital from January to August 2021 were consecutively recruited and further divided into the development group and the internal validation group. Perimenopausal women admitted to the Department of Cardiology of Hunan Provincial People's Hospital from March to June 2025 were selected as the external validation group. The inclusion criteria were as follows: (1) participants were perimenopausal women (40–60 years old); (2) participants consented to peripheral vein blood collection; (3) participants or family members had signed an informed consent form. The exclusion criteria were as follows: (1) patients with severe hematologic diseases, cardiac, renal, or hepatic functional diseases, and malignant tumors; (2) patients with infectious diseases, such as hepatitis B and tuberculosis; (3) patients who had recently used medications affecting hormone levels, lipid levels, and Hcy levels, or had taken fibrinolytic anticoagulant medications; and (4) women during lactation and pregnancy. Finally, a total of 687 women from the 2021 study group were enrolled and divided into a control group (*n* = 550) and an HHcy group (*n* = 137); the external validation group (enrolled from March to June 2025) included 63 participants. The study was ethically approved by the Medical Ethics Committee of Hunan Normal University (approval numbers: 2017034 and 2024061), and all study participants gave informed consent.

### 2.2 Diagnostic criteria

HHcy was defined as a fasting plasma Hcy level of 15 μmol/L or higher (9). Smoking was categorized as the regular smoking of at least one cigarette per day for a continuous period of at least six months (10). Alcohol consumption was defined as the intake of alcohol at least once per week for a minimum of six months (10). Tea consumption was defined as the consumption of tea at least three times per week for a minimum of six months (10). Macrosomia was defined as a fetal weight exceeding 4,000 g at any gestational age (11). Gestational diabetes mellitus was defined as glucose intolerance that emerges or is first detected during pregnancy (12). Pregnancy-induced hypertension (PIH) was defined as hypertension without proteinuria, occurring for the first time after 20 weeks of gestation, with a systolic blood pressure (SBP) greater than 140 mmHg and a diastolic blood pressure (DBP) greater than 90 mmHg (13). Irregular exercise was defined as engaging in physical activity once to three times per week or less, while regular exercise was defined as more than three times per week. The frequency of meat consumption was classified as follows: “Never” indicated less than one consumption per month, “Occasionally” indicated one to three meat meals per week, and “Often” indicated more than three meat meals per week.

### 2.3 Data collection

The demographic and lifestyle characteristics of the study participants were assessed using a questionnaire. The survey encompassed four primary domains: (1) demographic characteristics (including Age, Residential Area, Educational level, Menarche Age, Hypertension, and Menopause status); (2) pregnancy-related factors (including Age at First Birth, Macrosomia, PIH, and Gestational Diabetes Mellitus); (3) lifestyles (including Physical Activity, Smoking and Drinking habits, and Sleep Duration); and (4) diets (including consumption frequency of meat, eggs, vegetables, nuts, dairy products, fruits, soy products, alcohol, tea, and coffee). BMI (Body Mass Index) was calculated as weight (kg) divided by height squared (m^2^). We also collected the following biochemical indicators at admission: Triglycerides (TG), Total Cholesterol, Low-Density Lipoprotein (LDL), High-Density Lipoprotein (HDL), Alanine Aminotransferase, Aspartate Aminotransferase (AST), E2, Testosterone, Total Protein (TP), Albumin (ALB), Globulin (GLB), Albumin/Globulin ratio (A/G), Prothrombin Time (PT), Uric Acid (UA), Glomerular Filtration Rate (GFR), Serum Creatinine (Scr), Blood Urea Nitrogen, and Cystatin C (CysC).

### 2.4 Data pre-processing

The clinical research big data underwent rigorous cleaning, including the removal of outliers and imputation of missing values. Indicators with missing values over 20% were excluded from the analysis. Random-Forest Multiple Imputation (14) was used for handling missing predictor values. This method was implemented using the “mice” R package, which involved imputing the dataset five times; the average of these results constituted the final imputed values. The imputed dataset was then used for subsequent statistical analyses. The data were divided into a training set (70% of the data) and an internal validation set (30% of the data) for model training and evaluation. The classification model was trained using the training set, and its performance was assessed using the internal validation set and the external validation set.

### 2.5 Statistical methods

For quantitative variables, those obeying normality were described by mean ± standard deviation, and the *t*-test was used to compare the differences. Those not obeying normality were described by median and quartile intervals [M (P25, P75)], and the Mann–Whitney *U*-test was used to compare the differences. Qualitative variables were described by proportion (%), and differences were compared using the Chi-square (*χ*^2^) test or Fisher exact probability method.

Predictor selection and regularization were conducted utilizing LASSO regression analysis. The “glmnet” package in R was used to perform LASSO regression analysis to identify clinical characteristics that were significantly associated with the risk of HHcy. The variance inflation factor (VIF) was utilized to assess the severity of multicollinearity in the multivariate linear regression model, and significant variables were included in the multivariate logistic regression model to identify predictive factors. A VIF value < 10 could be regarded as the absence of high multicollinearity. Subsequently, Restricted Cubic Spline analysis was applied to assess the linear relationship between potential continuous predictors and HHcy risk before developing the multivariate logistic regression model, ensuring only variables with appropriate linear relationships were included, after which a multivariate logistic regression analysis was conducted to develop a predictive model capable of discriminating between HHcy and non-HHcy participants. The created model served as the basis for the development of a nomogram. The predictive efficacy of the model was evaluated in terms of discrimination, calibration, and clinical applicability by using the Receiver Operating Characteristic (ROC) curve, calibration curve, and clinical decision curve analysis (DCA). The “pROC” package in R was used to plot the ROC curves and calculate the area under the ROC curve (AUC) values. Data were analyzed using SPSS 26.0 statistical software and R 4.4.1 software. All statistical tests were two-sided, and a significance level of *P* < 0.05 was considered statistically significant.

## 3 Results

### 3.1 Study population characteristics

The dataset contained varying levels of missingness across different variables, ranging from 0.1% to 10.2% of total values. The variables with the highest percentage of missing values (10.2%) were Menarche Age, followed closely by Age at First Birth at 9.5%. Lower levels of missingness were observed for variables such as Smoking (0.1%), Alcohol Consumption (0.1%), and Egg Consumption Frequency (0.3%), as shown in Supplementary Figure S1. To properly handle missing data, this study used Random-Forest Multiple Imputation for imputation. A comparison of data distribution before and after imputation revealed no statistically significant differences between the original and imputed data (*P* > 0.05), as detailed in Supplementary Table S1.

The basic characteristics of patients with HHcy and controls are shown in Table 1*.* During the study period, we enrolled 687 eligible perimenopausal women with an average age of 52.73 years. According to the diagnostic criteria, 137 women were diagnosed with HHcy, and the prevalence was 19.94%. The training group had 481 women, with an average age of 52.77 years, and 97 women with HHcy (20.17%). The internal validation group had 206 women, with an average age of 52.64 years, and 40 women had HHcy (19.42%). No significant differences were observed between the two groups with regard to participants' characteristics (*P* > 0.05; Supplementary Table S2). Comparative analysis of basic characteristics between HHcy and non-HHcy groups revealed significant differences in Age, Education Level, Egg Consumption Frequency, PIH, Gestational Diabetes Mellitus, Hypertension, SBP, DBP, TG, LDL, TP, GLB, A/G, PT, UA, Scr, GFR, and CysC (*P* < 0.05).

**Table 1:** Patients’ characteristics of the enrolled population.

Variables	Non-HHcy (*n* = 550)	HHcy (*n* = 137)	*χ*^2^/Z	*P*
Age (years)	53.00 (50.00, 56.00)	54.00 (51.00, 57.00)	−2.332	0.020
Residential Area			0.147	0.701
Urban	267 (48.55%)	64 (46.72%)		
Rural	283 (51.45%)	73 (53.28%)		
Education Level			10.018	0.040
Illiterate	1 (0.18%)	3 (2.19%)		
Primary School	82 (14.91%)	25 (18.25%)		
Junior High School	243 (44.18%)	62 (45.26%)		
High School or Technical Secondary School	150 (27.27%)	34 (24.82%)		
College or Higher	74 (13.45%)	13 (9.49%)		
Menopause			3.191	0.074
Yes	414 (75.27%)	113 (82.48%)		
No	136 (24.73%)	24 (17.52%)		
Menarche Age (years)	14.00 (13.00, 14.00)	14.03 (13.00, 14.00)	−1.746	0.081
Age at First Birth (years)	24.00 (22.00, 25.00)	24.00 (22.00, 25.00)	−0.382	0.703
Physical Activity			1.753	0.416
Almost no exercise	209 (38.00%)	59 (43.07%)		
Irregular exercise	200 (36.36%)	42 (30.66%)		
Regular exercise	141 (25.64%)	36 (26.28%)		
Sedentary Time (hours)			0.245	0.970
1–3	89 (16.18%)	21 (15.33%)		
3–5	303 (55.09%)	74 (54.01%)		
5–7	126 (22.91%)	34 (24.82%)		
>7	32 (5.82%)	8 (5.84%)		
Sleep Duration (hours)	7 (6, 8)	7 (5, 8)	−1.249	0.212
Smoking			0.000	1.000
Current smoker	12 (2.18%)	3 (2.19%)		
Former smoker	4 (0.73%)	1 (0.73%)		
Never smoked	534 (97.09%)	133 (97.08%)		
Alcohol Consumption				0.377*
Yes	13 (2.36%)	5 (3.65%)		
No	537 (97.64%)	132 (96.35%)		
Coffee Consumption				0.133*
Yes	11 (2.00%)	0 (0.00%)		
No	539 (98.00%)	137 (100.00%)		
Tea Consumption			3.670	0.055
Yes	104 (18.91%)	36 (26.28%)		
No	446 (81.09%)	101 (73.72%)		
Macrosomia			3.661	0.160
Yes	45 (8.18%)	17 (12.41%)		
No	486 (88.36%)	118 (86.13%)		
Uncertain	19 (3.45%)	2 (1.46%)		
PIH			7.292	0.026
Yes	15 (2.73%)	6 (4.38%)		
No	427 (77.64%)	117 (85.40%)		
Uncertain	108 (19.64%)	14 (10.22%)		
Gestational Diabetes Mellitus			6.629	0.036
Yes	2 (0.36%)	0 (0.00%)		
No	438 (79.64%)	122 (89.05%)		
Uncertain	110 (20.00%)	15 (10.95%)		
Hypertension			12.987	<0.001
Yes	250 (45.45%)	39 (28.47%)		
No	300 (54.55%)	98 (71.53%)		
BMI (kg/m²)	23.31 (21.48, 25.68)	23.12 (21.35, 25.05)	−0.528	0.597
Heart Rate (bpm)	78 (70, 85)	76 (68, 85)	−0.682	0.495
Abdominal Circumference (cm)	85 (80, 90)	85 (80, 90)	−0.249	0.803
SBP (mmHg)	133 (119, 146)	137 (125, 154)	−2.931	0.003
DBP (mmHg)	80 (72, 90)	87 (78, 93)	−3.631	<0.001
Meat Consumption Frequency			0.551	0.759
Never	24 (4.36%)	8 (5.84%)		
Occasionally	356 (64.73%)	88 (64.23%)		
Often	170 (30.91%)	41 (29.93%)		
Types of Meat Consumed			2.954	0.565
Pork	454 (82.55%)	117 (85.40%)		
Chicken, Duck	45 (8.18%)	10 (7.30%)		
Beef, Lamb	5 (0.91%)	0 (0.00%)		
Fish	18 (3.27%)	6 (4.38%)		
Other	28 (5.09%)	4 (2.92%)		
Egg Consumption Frequency (times/week)			9.653	0.002
≤3	407 (74.00%)	88 (64.23%)		
>3	143 (26.00%)	54 (39.42%)		
Soy Product Consumption Frequency (times/week)			0.074	0.785
≤3	506 (92.00%)	127 (92.70%)		
>3	44 (8.00%)	10 (7.30%)		
Dairy Product Consumption Frequency (times/week)			1.958	0.162
≤3	486 (88.36%)	115 (83.94%)		
>3	64 (11.64%)	22 (16.06%)		
Fruit Consumption Frequency (times/week)			1.074	0.300
≤3	320 (58.18%)	73 (53.28%)		
>3	230 (41.82%)	64 (46.72%)		
Vegetable Consumption Frequency (times/week)				1.000*
≤3	12 (2.18%)	3 (2.19%)		
>3	538 (97.82%)	134 (97.81%)		
Nut Consumption Frequency (times/week)			1.192	0.275
≤3	507 (92.18%)	130 (94.89%)		
>3	43 (7.82%)	7 (5.11%)		
Use of Health Supplements			1.768	0.184
Yes	90 (16.36%)	29 (21.17%)		
No	460 (83.64%)	108 (78.83%)		
E2 (pg/ml)	22.30 (14.62, 30.65)	20.60 (13.73, 32.90)	−0.004	0.997
Testosterone (nmol/ml)	0.36 (0.28, 0.42)	0.36 (0.22, 0.45)	−0.681	0.496
TG (mmol/L)	1.48 (1.05, 2.08)	1.62 (1.30, 2.28)	−2.480	0.013
Total Cholesterol (mmol/L)	4.45 (3.81, 5.14)	4.59 (4.03, 5.32)	−1.878	0.060
LDL (mmol/L)	2.62 (2.09, 3.21)	2.88 (2.32, 3.49)	−2.457	0.014
HDL (mmol/L)	1.22 (1.05, 1.42)	1.16 (0.96, 1.44)	−1.867	0.062
Alanine Transaminase (U/L)	17.40 (12.60, 25.10)	17.00 (12.00, 25.90)	−0.370	0.712
AST (U/L)	19.95 (17.02, 24.47)	21.00 (17.00, 25.80)	−0.852	0.394
TP (g/L)	64.70 (60.80, 68.30)	67.33 (63.00, 71.50)	−4.207	<0.001
ALB (g/L)	40.70 (38.50, 43.10)	42.00 (38.63, 44.00)	−1.919	0.055
GLB (g/L)	23.80 (21.33, 26.30)	25.50 (22.70, 28.10)	−4.174	<0.001
A/G (Ratio)	1.72 (1.55, 1.93)	1.61 (1.45, 1.87)	−3.144	0.002
PT (s)	10.10 (9.40, 10.90)	11.00 (10.00, 11.80)	−7.167	<0.001
UA (*μ*mol/L)	248.70 (5.73, 324.38)	306.00 (206.00, 401.00)	−5.172	<0.001
Scr (μmol/L)	54.26 (47.41, 62.98)	68.00 (56.00, 95.00)	−8.620	<0.001
Blood Urea Nitrogen (mmol/L)	5.62 (4.37, 241.90)	6.54 (4.60, 23.80)	−0.738	0.461
GFR (ml/min/1.73 m^2^)	103.76 (95.52, 109.98)	81.20 (55.10, 99.80)	−9.663	<0.001
CysC (mg/L)	0.80 (0.62, 0.98)	1.05 (0.92, 1.32)	−9.481	<0.001

* Using Fisher's exact test. SBP, systolic blood pressure; DBP, diastolic blood pressure; BMI, body mass index; PIH, pregnancy-induced hypertension; E2, estradiol; TG, triglycerides; LDL, low-density lipoprotein; HDL, high-density lipoprotein; AST, aspartate aminotransferase; TP, total protein; ALB, albumin; GLB, globulins; A/G, albumin/globulin ratio; PT, prothrombin time; UA, uric acid; Scr, serum creatinine; GFR, glomerular filtration rate; CysC, cystatin C.

### 3.2 Variable selection

**Figure 1:**
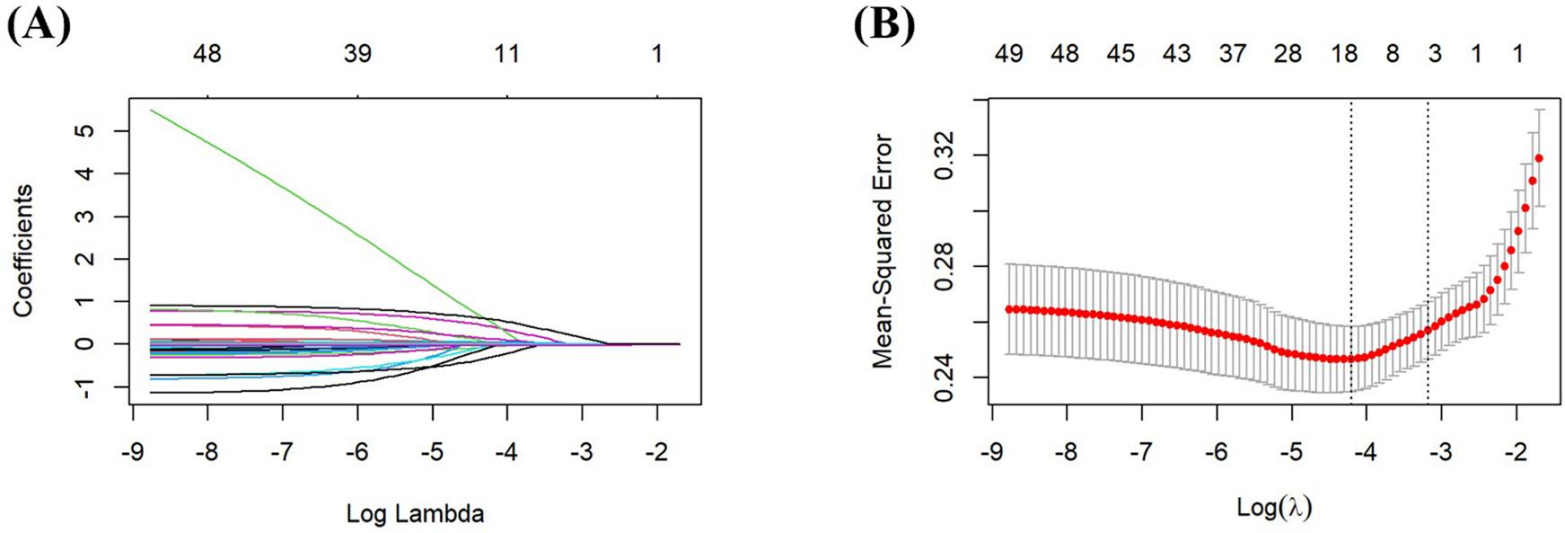
Screening predictors based on LASSO regression. **(A)** Ten-fold cross-validation was performed to determine the optimal value of the LASSO regression-related tuning parameter (lambda). **(B)** The coefficient profiles of the variables incorporated in the LASSO regression analysis were plotted against the logarithm of the lambda sequence.

LASSO regression analysis was performed to identify the potential predictive factors. As the penalty parameter *λ* was adjusted, the number of variables included in the model decreased progressively. A 10-fold cross-validation was performed, and the lambda value corresponding to the minimum (*λ*.min) was determined to be 0.015 (Figure 1). Initial LASSO regression analysis of 49 potential variables identified 16 predictors with non-zero coefficients, including Education Level, Menarche Age, Egg Consumption Frequency, Nut Consumption Frequency, Coffee Consumption, PIH, Gestational Diabetes Mellitus, Heart Rate, Testosterone, LDL, HDL, AST, TP, PT, GFR, and CysC.

### 3.3 Prediction model establishment using selected factors

The multicollinearity test revealed no statistically significant correlations among the variables (Supplementary Table S3). Specifically, the VIFs value of all 49 initial predictor variables ranged from 1.03 to 2.87, with all values well below the widely accepted threshold of 10.

The 16 selected variables were used as independent predictors, with HHcy occurrence as the dependent variable. Multifactorial binary logistic regression analysis showed that Egg Consumption Frequency [odds ratio (OR) = 0.545, 95% CI = 0.301–0.987, *P* < 0.05], LDL (OR = 1.419, 95% CI = 1.017–1.978, *P* < 0.05), TP (OR = 1.071, 95% CI = 1.026–1.117, *P* < 0.05), and CysC (OR = 9.378, 95% CI = 4.582–19.193, *P* < 0.001) were identified as predictors for HHcy (*P* < 0.05; Table 2*)*. Multifactorial binary logistic regression analysis was conducted with further adjustment of age and E2, and the results were consistent with the main finding (Supplementary Table S4).

**Table 2:** Multifactorial binary logistic regression analysis of independent risk factors based on LASSO.

Variables	B	SE	Wald	P	OR (95% CI)
Education Level (ref: College or Higher)					
Illiterate	2.630	1.595	2.718	0.099	13.874 (0.609,316.229)
Primary School	−0.013	0.512	0.001	0.980	0.987 (0.362,2.693)
Junior High School	−0.031	0.438	0.005	0.944	0.970 (0.411,2.287)
High School or Technical Secondary School	−0.502	0.479	1.102	0.294	0.605 (0.237,1.546)
Menarche Age	0.248	0.379	0.428	0.513	1.206 (0.986,1.475)
Egg Consumption Frequency (ref: More than three times)	−0.607	0.303	4.008	0.045	0.545 (0.301,0.987)
Nut Consumption Frequency (ref: More than three times)	0.694	0.600	1.337	0.247	2.002 (0.617,6.495)
Coffee Consumption (ref: Yes)	−20.343	12303.794	0.000	0.999	0.000
PIH (ref: Uncertain)					
Yes	−19.026	40192.746	0.000	1.000	0.000
No	−18.543	40192.746	0.000	1.000	0.000
Gestational Diabetes Mellitus (ref: Uncertain)					
Yes	1.796	48716.670	0.000	1.000	6.028
No	20.006	40192.746	0.000	1.000	488149226.419
Heart Rate	−0.011	0.008	2.058	0.151	0.989 (0.974,1.004)
Testosterone	1.555	0.878	3.138	0.077	4.737 (0.847,26.480)
LDL	0.350	0.170	4.249	0.039	1.419 (1.017,1.978)
HDL	−0.793	0.489	2.631	0.105	0.453 (0.174,1.180)
AST	0.011	0.007	2.548	0.110	1.011 (0.997,1.026)
TP	0.068	0.022	9.931	0.002	1.071 (1.026,1.117)
PT	0.042	0.033	1.637	0.201	1.043 (0.978,1.113)
GFR	0.000	0.004	0.000	0.995	1.000 (0.993,1.007)
CysC	2.238	0.365	37.521	0.000	9.378 (4.582,19.193)

PIH, pregnancy-induced hypertension; LDL, low-density lipoprotein; HDL, high-density lipoprotein; AST, aspartate aminotransferase; TP, total protein; PT, prothrombin time; GFR, glomerular filtration rate; CysC, cystatin C.

**Figure 2:**
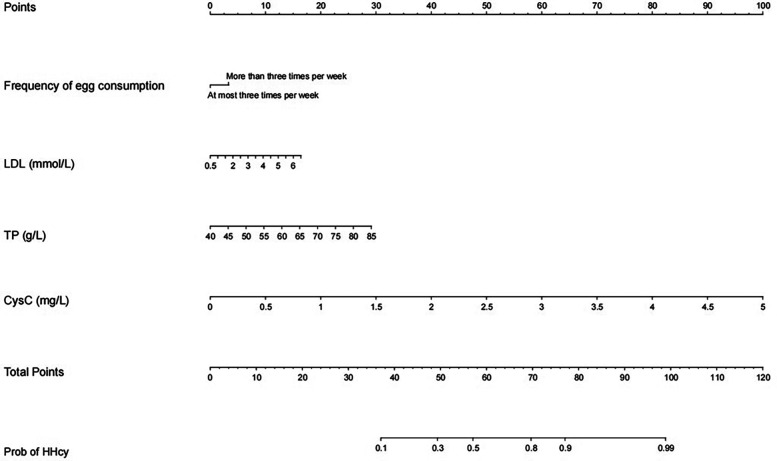
Nomogram model based on LASSO logistic regression. The nomogram represents the prediction probability of HHcy, ranging from 0 to 120. For each predictive, a vertical line is drawn to the point axis, and the corresponding point is noted down. The scores of each predictor are summed. The total score corresponding to the predicted occurrence of probative variability of HHcy is provided at the bottom of the nomogram.

Note: For the categorical variable “Egg Consumption Frequency” in the equation, the assignment is defined as follows: 1 represents egg consumption frequency < 3 times/week, and 0 represents egg consumption frequency ≥ 3 times/week.

Analysis of the continuous variables in the model revealed linear relationships between LDL, TP, CysC, and the prevalence of HHcy. Since the logistic regression model is a linear model in terms of logit, these linear relationships satisfy the basic conditions for modeling using logistic regression analysis (Supplementary Figure S2). A diagnostic model for the training group was constructed based on these four independent variables, visualized using a nomogram (Figure 2). These variables were incorporated into the development of the nomogram for HHcy risk prediction. The underlying regression equation of this nomogram is:

Logit(P)=−12.51−0.61×(EggConsumptionFrequency)+0.35×(LDL)+0.07×(TP)+2.24×(CysC)

Each variable's values were assigned scores on the scale axis based on the magnitude of their regression coefficients. The sum of individual scores yielded a total score, and the probability of HHcy occurrence was calculated along the total score scale axis. To demonstrate the clinical utility of the nomogram, a practical example is provided as follows: For a hypothetical perimenopausal patient with an egg consumption frequency of more than 3 times per week, an LDL level of 6 mmol/L, a TP level of 70 g/L, and a CysC level of 1 mg/L, first locate the patient's specific values for each variable on the corresponding variable axes of the nomogram, then draw a vertical line upward from each variable value to the “points axis” to obtain the component score for each variable—specifically, approximately 5 points for egg consumption frequency, approximately 15 points for LDL at the concentration of 6 mmol/L, approximately 20 points for TP at the concentration of 70 g/L, and approximately 20 points for CysC at the concentration of 1 mg/L—subsequently sum these component scores to calculate the total score (5 points for egg consumption frequency + 15 points for LDL + 20 points for TP + 20 points for CysC = 60 points), and finally draw a vertical line downward from the total score (60 points) to the “probabilit*y* axis,” where the corresponding value represents the predicted probability of HHcy for this patient, approximately 50%–60% in this case.

### 3.4 Internal evaluation of the prediction model: accuracy and calibration

**Figure 3:**
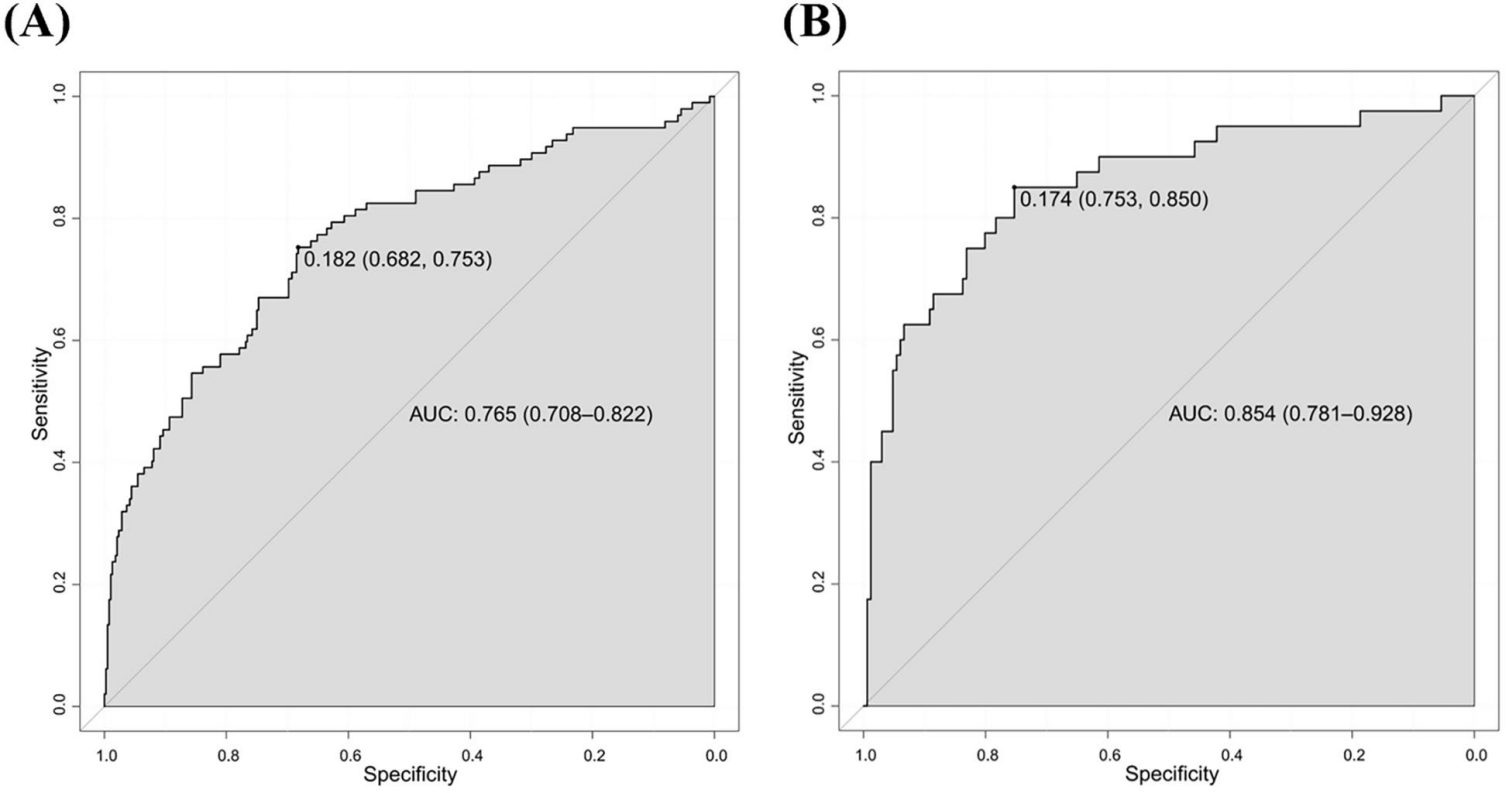
ROC curves for predicting risk of HHcy in perimenopausal women **(A)** training set **(B)** internal validation set.

**Figure 4:**
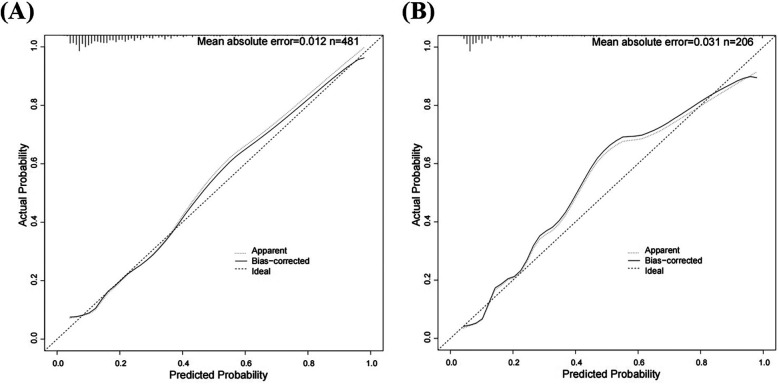
Calibration curves for predicting risk of HHcy in perimenopausal women **(A)** training set **(B)** internal validation set.

**Figure 5:**
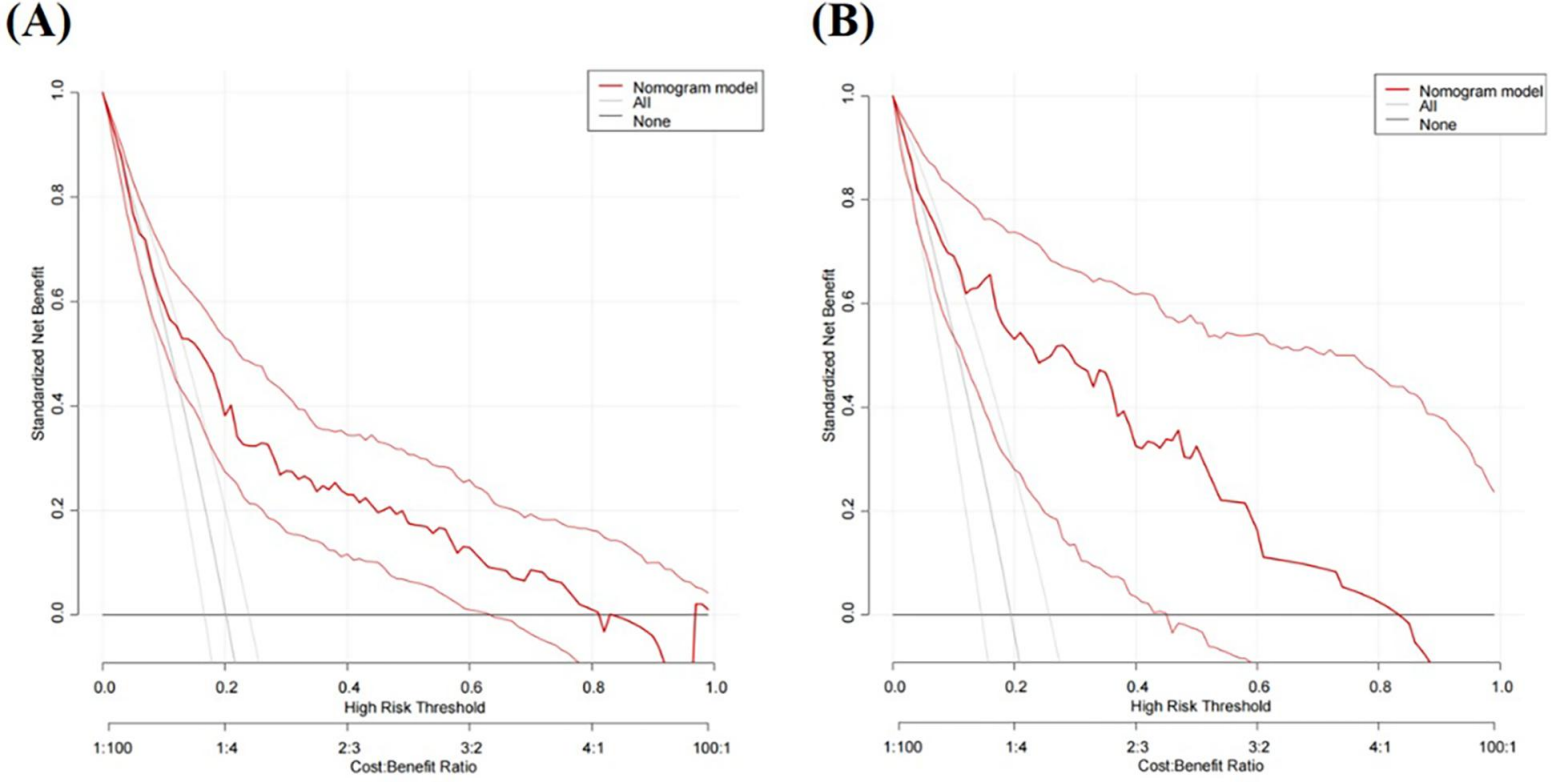
DCA curves for predicting risk of HHcy in perimenopausal women **(A)** training set **(B)** internal validation set.

We initially plotted the ROC curve of the model in the training set (Figure 3A), with an AUC of 0.765 (95% CI = 0.708–0.822). On this curve, when the specificity reached 0.682, the corresponding sensitivity was 0.753, which reflects the trade-off between sensitivity and specificity of the model in the training set and indicates the good clinical diagnostic performance of the model. The calibration curve suggested that the mean absolute error (MAE) between the predicted and actual values was 0.012 (Figure 4A), indicating that the predicted risk closely aligns with the actual risk. As the nomogram model was constructed based on the training set, we evaluated and validated the model in the validation set, resulting in an AUC of 0.854 (95% CI = 0.781–0.928) (Figure 3B). For the internal validation set ROC curve, when the specificity was 0.753, the corresponding sensitivity was 0.850. The calibration curve showed that the MAE between the predicted values and the actual values was 0.031 (Figure 4B). The DCA results of the training set (Figure 5A) and the internal validation set (Figure 5B) showed that the predictive model occupied a high position on the decision curve. The DCA curves clearly indicated that within a specific “high-risk threshold” range, the performance of the nomogram model (the red curve) was superior to both the “intervene all” (the gray curve) and “intervene none” (the black line) strategies. In particular, when the threshold probability fell within the interval of 0.2–0.8, the standardized net benefit of the model was significantly higher, which fully demonstrated that the model had higher net benefit and clinical application value.

### 3.5 External validation of the prediction model

**Figure 6:**
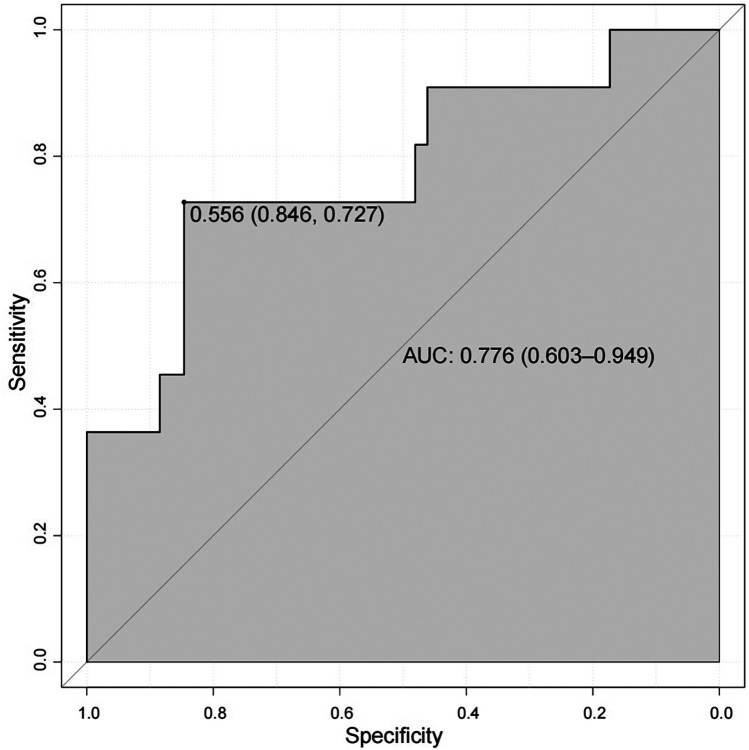
ROC curve for external validation of the HHcy prediction model in perimenopausal women.

From March to June 2025, 63 perimenopausal women were selected from those hospitalized in the Department of Cardiology of Hunan Provincial People's Hospital during this period, all of whom met the specific inclusion and exclusion criteria of the study. Among them, 11 perimenopausal women were diagnosed with HHcy, accounting for 17.46% of the total study participants. ROC curve analysis (Figure 6) showed that the AUC of the nomogram model for predicting HHcy risk in the external validation group of perimenopausal women was 0.776 (95% CI = 0.603–0.949); specifically, when the specificity reached 0.846, the corresponding sensitivity was 0.727. In addition, the MAE between the predicted values and actual values of the model was 0.055.

## 4 Discussion

In this study, we found that the frequency of egg consumption, LDL, TP, and CysC were significant predictors of HHcy in perimenopausal women using LASSO regression, demonstrating high predictive accuracy and clinical applicability. The AUC for the predictive model was 0.765, and the internal validation AUC was 0.854. The Hosmer-Lemeshow goodness-of-fit calibration curve showed that the MAEs of the training and internal validation sets were 0.012 and 0.031, respectively. For external validation, ROC curve analysis showed the nomogram had an AUC of 0.776 (95% CI = 0.603–0.949) for predicting HHcy risk in the external validation group, with a MAE of 0.055 between the model's predicted values and actual outcomes.

The findings of this study on HHcy in perimenopausal women are highly consistent with existing research conclusions regarding Hcy metabolism and its clinical significance, while also extending such knowledge. The prevalence of HHcy in this study was 19.94%, a figure consistent with the results of studies on different populations. For example, a cross-sectional survey covering 10,511 middle-aged and elderly individuals in China showed that the prevalence of HHcy in this population was 22.00% (9); another study involving Japanese patients with stroke complicated by chronic kidney disease (CKD) reported that the prevalence of HHcy among its subjects was 18.50% (15).Although there are obvious differences in population characteristics between the above two studies and this one, the prevalence of HHcy in perimenopausal women in this study falls exactly within the numerical range of the existing research results. This finding suggests that the epidemiological characteristics of HHcy in perimenopausal women are not completely independent of the metabolic laws of the general adult population, but share certain commonalities with them. At the same time, the prevalence data of this study also provide a key reference for subsequent comparisons of Hcy level distribution among women in different physiological stages and different health statuses, filling the partial gap in epidemiological data on HHcy in women during the special physiological stage of perimenopause.

The frequency of egg consumption was considered an important predictor in our study. Specifically, perimenopausal women who consumed eggs no more than three times per week were found to have a 0.545-fold risk of developing HHcy compared to those who consumed eggs more frequently (more than three times per week). Few studies have confirmed a direct link between egg intake and elevated Hcy levels, and existing evidence remains inconsistent. On one hand, the association between excessive egg intake and elevated Hcy levels is biologically plausible. First, eggs are an important source of methionine, which is metabolized in the body by transmethylation to form homocysteine (16). Excessive methionine intake leads to HHcy, which is a causative agent of cardiovascular disease in humans (17). Furthermore, excessive consumption of egg yolks can increase LDL levels (18). Changes in LDL levels also affect Hcy concentrations. On the other hand, a 2011 randomized controlled trial found that among participants with type 2 diabetes or impaired glucose tolerance, there was no significant association between egg consumption (two eggs per day) and Hcy levels (19). This suggests that the association between egg intake and HHcy may be influenced by glucose metabolism status while the participants in our study were mainly perimenopausal women with normal glucose metabolism. Under the state of glucose metabolic homeostasis, the methionine metabolism pathway is more susceptible to the regulation of dietary methionine intake, which may thereby strengthen the association between egg consumption and HHcy.

Although the mechanism by which LDL influences Hcy concentrations is not fully understood, LDL and its oxidized form (OxLDL) are known to accumulate in the arterial intima, triggering adaptive immunity and initiating a cascade of events that can lead to atherosclerosis and endothelial dysfunction (20). Endothelial dysfunction shares risk factors with HHcy, such as increased oxidative stress and reduced nitric oxide bioavailability. Additionally, there is a significant genetic component involved in the regulation of reactive oxygen species, Hcy levels, and atherogenesis (21). The increased correlation between HHcy and dyslipidemia in perimenopausal women may be attributed to changes in metabolism, hormonal levels, and lifestyle factors. However, it is crucial to acknowledge that this association might be influenced by unmeasured subclinical inflammation. As a common underlying condition in metabolic disorders, chronic low-grade inflammation can both disrupt lipid metabolism and promote Hcy production via pathways like oxidative stress or impaired enzyme activity in Hcy metabolism (22). Therefore, the association between LDL and HHcy may not solely reflect direct biological interactions but could also incorporate indirect effects of inflammation acting as a shared driver. The total protein in the serum is made up of two main categories: ALB and GLB. These components are important for assessing nutritional status and diagnosing various diseases. Methionine is regenerated via the retrieval of a methyl group from 5-methyltetrahydrofolate, a process that converts 5-methyltetrahydrofolate to tetrahydrofolate; tetrahydrofolate is subsequently converted back to 5-methyltetrahydrofolate by methylenetetrahydrofolate reductase. This process is called remethylation. Alternatively, Hcy can follow the transsulfuration route, where through cystathionine-beta-synthase, it is irreversibly converted into cystathionine, a precursor of cysteine, glutathione, and other substances that are finally excreted in the urine. HHcy results from inhibition of the remethylation route, or inhibition or saturation of the transsulfuration pathway (23). Higher levels of TP may imply a more active methylation reaction *in vivo*, thus affecting Hcy metabolism. However, it is important to note that confounding factors may exist in the association between TP levels and HHcy: TP levels can indirectly reflect underlying nutritional status. For instance, mild malnutrition may simultaneously reduce serum TP synthesis and impair Hcy metabolism by limiting the intake of critical micronutrients essential for Hcy clearance (24). Consequently, the observed association between TP and HHcy may not represent a direct causal relationship but could, to some extent, be driven by unmeasured nutritional factors.

Serum CysC was identified as the most significant predictor of HHcy in perimenopausal women in this study. The kidney is one of the important sites for Hcy metabolism. Hcy levels are closely associated with renal function. Previous studies have indicated that Hcy is elevated in patients with CKD and increases as the disease progresses (25). Numerous studies have established an association between Hcy and renal function indicators (26). Notably, CysC, Scr, and GFR share overlapping biological correlations and clinical significance, while CysC also exhibits unique advantages (27). Biologically, all three are linked to glomerular filtration function. Clinically, their significance overlaps in that all three are used to assess renal function and predict renal-related complications. However, serum Scr-based GFR has limitations due to its dependence on muscle mass, dietary intake, and tubular secretion. CysC is less influenced by these factors, potentially offering a more accurate reflection of kidney function, especially in certain populations such as the elderly or those with reduced muscle mass (28). CysC, a cysteine protease inhibitor, is ubiquitous in body fluids and nucleated cells throughout the human body (29). Regarding its potential role as an inflammatory marker, preclinical studies suggest CysC may influence Hcy metabolism through inhibition of cystathionine *γ*-lyase, an enzyme involved in Hcy catabolism. Additionally, both CysC and Hcy have been linked to pro-inflammatory pathways, including oxidative stress and endothelial dysfunction, which could create bidirectional relationships that are difficult to parse in observational data (30). However, these mechanistic links remain to be fully validated in clinical settings, and our study design cannot definitively establish causality.

LASSO regression analysis is a widely used statistical method for feature selection. It constructs a penalty function by compressing the regression coefficients. The advantages of this method lie in avoiding overfitting and extracting significant features effectively. Besides, LASSO is more advantageous in situations where there are various clinical parameters and a limited sample size (31). In addition, it outperforms stepwise logistic regression, ridge regression, and elastic net, thanks to its features of sparse variable selection (directly setting the coefficients of irrelevant variables to 0) and mitigation of multicollinearity (32). Furthermore, LASSO-logistic regression is an optimized extension of the traditional linear model framework. It not only retains the core advantages of traditional linear models—such as the ability to quantify the association between variables and outcomes and good adaptability to moderate sample sizes—but also addresses the limitations of traditional linear models in handling multiple variables through coefficient shrinkage. Ultimately, LASSO identified 4 independent predictive factors for HHcy, meeting the clinical demand for a parsimonious and stable model. LASSO regression identified fewer variables than expected based on clinical experience, which can be attributed to several straightforward reasons. This study focused specifically on perimenopausal women aged 40–60 years, and several cohort-specific characteristics may help explain the variable selection outcomes. First, regarding BMI—a factor often considered clinically relevant—this study's perimenopausal women exhibited a relatively narrow BMI distribution, which likely reduced its ability to serve as a distinct predictive marker for HHcy. Second, for traditional risk factors like smoking, the number of smokers among the perimenopausal women was particularly small; this limited sample size for smoking status may have weakened its statistical association with the outcome, leading to its exclusion from the final model (33). Furthermore, the unique hormonal fluctuations inherent to women in this specific age group may also have modulated the relationships between potential predictors and HHcy, further influencing which variables remained in the model. Finally, it is important to note that differences in the datasets and samples used—including variations in overall sample size, participant origin, and data collection timing—can also introduce variability in results, and these factors may have contributed to the final set of selected predictors as well.

In this study, we established a predictive statistical model to assess the risk of HHcy in perimenopausal women, and the nomogram not only visually presents the independent risk factors identified in multivariate regression analysis but also enables prediction through simple graphics. This tool will help doctors to accurately predict the risk of HHcy and provide a powerful tool for clinical management. To further enhance its accessibility and practicality in primary care settings, we plan to develop a user-friendly web-based calculator based on this nomogram, which will allow clinicians to automatically calculate HHcy risk by inputting patients’ egg consumption frequency, LDL levels, TP levels, and CysC levels. Concurrently, we will conduct a pilot application in 3 hospitals to verify its usability and predictive consistency in real-world clinical scenarios. Notably, although our study population focuses on perimenopausal women aged 40–60 years, there is still objective heterogeneity within this group. As supported by relevant studies in the field (34), this heterogeneity may contribute to differential predictive performance of the nomogram across subgroups of this population. Meanwhile, the modifiable risk factors identified by the model highlight the relevance of analyzing causal associations between intervention measures and HHcy outcomes. Within the framework of target trial emulation (35), methods like propensity score matching and inverse probability weighting offer approaches to more reasonably control confounding factors. This can strengthen the reliability of evidence when evaluating the link between modifying these risk factors and changes in HHcy risk, and further provide implications for guiding HHcy management in perimenopausal women.

The strengths of this paper are, first, that LASSO regression was used for variable screening, with its most significant advantage over traditional univariate analysis being the ability to automate variable selection. Secondly, we included complete information, including demographic characteristics, pregnancy-related factors, lifestyles, and diets. Finally, our model was validated and showed good accuracy and stability. It is targeted at perimenopausal women and can provide some guidance for the prevention of HHcy in this special population. Several limitations of this study need to be recognized. This study is retrospective, with selection bias (hospital recruitment overrepresenting perimenopausal women with chronic conditions, underrepresenting healthy ones) and information bias (retrospective self-reported dietary data may cause recall bias); however, the real-world model has in-hospital clinical value, and future prospective studies should validate it in community cohorts while including nutritional biomarkers (folate, vitamin B6, B12) detection to better control nutritional confounding. Additionally, despite the use of LASSO regression for variable selection, potential overfitting risk remains due to limited sample size and initial multiple predictors, and subsequent studies will expand sample size, optimize criteria, and use stricter validation to boost model stability. Finally, the generalizability of the study is limited because it was conducted in only one region of China, and future studies should expand it to other regions.

## 5 Conclusion

This study identified independent risk factors (including egg consumption frequency, LDL, TP, and CysC) for HHcy and developed a predictive risk model for perimenopausal women using LASSO regression combined with multifactorial binary logistic regression methods, showing good diagnostic efficacy and calibration. Based on these findings, regular monitoring of these factors can aid in the early detection and reduction of HHcy. The clinical implementation of this tool may contribute to reducing the prevalence of cardiovascular diseases by identifying individuals with early-stage HHcy and enabling targeted interventions.

## Data Availability

The original contributions presented in the study are included in the article/Supplementary Material, further inquiries can be directed to the corresponding author/s.
