# Supplementary material for: Precision prediction of hyperhomocysteinemia development in perimenopausal women using LASSO regression
Source: Front Reprod Health. 2025 Oct 9;7:1670141. doi: 10.3389/frph.2025.1670141 (PMC12546042; doi:10.3389/frph.2025.1670141)
Supplement: Supplementary file 1 [file Datasheet1.pdf]

Supplementary Table 1 Comparison of Data Distribution Between Original Data and Imputed Data

Variables	Original Data	Imputed Data	P
Menarche Age (years)	14.00 (13.00,14.00)	14.00 (13.00,14.00)	0.972^{ns}
Age at First Birth (years)	24.00 (22.00,25.00)	24.00 (22.00,25.00)	0.971^{ns}
Sleep Duration (hours)	7.00 (5.00,8.00)	7.00 (6.00,8.00)	0.163^{ns}
BMI (kg/m²)	22.94 (21.37,25.00)	23.31 (21.48,25.39)	0.257^{ns}
Heart Rate (bpm)	77.00 (69.00,86.00)	77.00 (70.00,85.00)	0.937^{ns}
Abdominal Circumference (cm)	84.00 (80.00,90.00)	85.00 (80.00,90.00)	0.943^{ns}
SBP (mmHg)	133.00 (120.00,147.00)	134.00 (120.00,148.00)	0.905^{ns}
DBP (mmHg)	80.00 (72.00,90.00)	81.00 (73.00,90.00)	0.994^{ns}
E2 (pg/mL)	21.60 (14.60,32.10)	22.00 (14.60,31.50)	0.658^{ns}
Testosterone (nmol/mL)	0.36 (0.27,0.42)	0.36 (0.27,0.42)	0.932^{ns}
TG (mmol/L)	1.48 (1.04,2.08)	1.52 (1.10,2.14)	0.986^{ns}
Total Cholesterol (mmol/L)	4.39 (3.78,5.16)	4.50 (3.86,5.20)	0.992^{ns}
LDL (mmol/L)	2.59 (2.08,3.23)	2.64 (2.12,3.25)	0.979^{ns}
HDL (mmol/L)	1.20 (1.03,1.41)	1.21 (1.03,1.43)	0.981^{ns}
Alanine Transaminase (U/L)	17.00 (12.40,26.20)	17.30 (12.50,25.20)	0.997^{ns}
AST (U/L)	20.26 (17.00,25.28)	20.07 (17.00,24.60)	0.994^{ns}
TP (g/L)	64.40 (60.70,68.40)	65.10 (61.00,69.08)	0.982^{ns}
ALB (g/L)	40.70 (38.40,43.30)	40.90 (38.50,43.40)	0.974^{ns}
GLB (g/L)	23.80 (21.20,26.33)	23.90 (21.50,26.70)	0.987^{ns}
A/G (Ratio)	1.71 (1.54,1.95)	1.70 (1.52,1.92)	0.990^{ns}
PT (s)	10.20 (9.40,11.00)	10.20 (9.50,11.10)	0.949^{ns}
UA (µmol/L)	250.00 (5.82,329.50)	260.00 (6.26,337.00)	0.985^{ns}

Continued Supplementary Table 1 Comparison of Data Distribution Between Original Data and Imputed Data

**Variables**	**Original Data**	**Imputed Data**	**P**
Scr (μmol/L)	55.97 (48.89,65.52)	55.80 (48.80,65.97)	0.988^{ns}
Blood Urea Nitrogen (mmol/L)	5.86 (4.43,238.10)	5.75 (4.40,232.00)	0.986^{ns}
GFR (mL/min/1.73m²)	102.30 (88.50,108.90)	102.29 (87.29,108.90)	0.519^{ns}
CysC (mg/L)	0.86 (0.65,1.02)	0.87 (0.65,1.04)	0.911^{ns}
Residence Area			0.978^{ns}
Urban	330 (48.10%)	331 (48.18%)	
Rural	356 (51.90%)	356 (51.82%)	
Menopause			1.000^{ns}
Yes	526 (76.68%)	527 (76.71%)	
No	160 (23.32%)	160 (23.29%)	
Use of Health Supplements			0.941^{ns}
Yes	119 (17.47%)	119 (17.32%)	
No	562 (82.53%)	568 (82.68%)	
Alcohol Consumption			0.986^{ns}
Yes	18 (2.64%)	18 (2.62%)	
No	665 (97.36%)	669 (97.38%)	
Coffee Consumption			0.992^{ns}
Yes	11 (1.60%)	11 (1.60%)	
No	673 (98.40%)	676 (98.40%)	
Tea Consumption			0.945^{ns}
Yes	140 (20.53%)	140 (20.38%)	
No	542 (79.47%)	547 (79.62%)	
Hypertension			1.000^{ns}
Yes	289 (42.07%)	289 (42.07%)	
No	398 (57.93%)	398 (57.93%)	
No	398 (57.93%)	398 (57.93%)	

Continued Supplementary Table 1 Comparison of Data Distribution Between Original Data and Imputed Data

**Variables**	**Original Data**	**Imputed Data**	**P**
Education Level			1.000^{ns}
Illiterate	4 (0.58%)	4 (0.58%)	
Primary School	106 (15.63%)	107 (15.57%)	
Junior High School	302 (44.54%)	305 (44.40%)	
High School or Technical Secondary School	183 (26.99%)	184 (26.78%)	
College or Higher	83 (12.24%)	87 (12.66%)	
Physical Activity			0.998^{ns}
Almost no exercise	267 (39.03%)	268 (39.01%)	
Irregular exercise	240 (35.09%)	242 (35.23%)	
Regular exercise	177 (25.88%)	177 (25.76%)	
Sedentary Time (hours)			0.989^{ns}
1-3	110 (16.39%)	110 (16.01%)	
3-5	362 (53.95%)	377 (54.88%)	
5-7	159 (23.70%)	160 (23.29%)	
>7	40 (5.96%)	40 (5.82%)	
Meat Consumption Frequency			0.997^{ns}
Never	32 (4.72%)	32 (4.66%)	
Occasionally	437 (64.45%)	444 (64.63%)	
Often	209 (30.83%)	211 (30.71%)	
Types of Meat Consumed			0.996^{ns}
Pork	537 (82.24%)	571 (83.11%)	
Chicken, Duck	55 (8.42%)	55 (8.00%)	
Beef, Lamb	5 (0.77%)	5 (0.73%)	
Fish	24 (3.68%)	24 (3.49%)	
Other	32 (4.90%)	32 (4.66%)	
Egg Consumption Frequency (times/week)			0.973^{ns}
≤3	488 (71.24%)	490 (71.32%)	
>3	197 (28.76%)	197 (28.68%)	

Continued Supplementary Table 1 Comparison of Data Distribution Between Original Data and Imputed Data

Variables	Original Data	Imputed Data	P
Soy Product Consumption Frequency (times/week)			0.932^{ns}
≤3	632 (92.26%)	633 (92.14%)	
>3	53 (7.74%)	54 (7.86%)	
Dairy Product Consumption Frequency (times/week)			0.959^{ns}
≤3	596 (87.39%)	601 (87.48%)	
>3	86 (12.61%)	86 (12.52%)	
Fruit Consumption Frequency (times/week)			0.981^{ns}
≤3	390 (57.27%)	393 (57.21%)	
>3	291 (42.73%)	294 (42.79%)	
Vegetable Consumption Frequency (times/week)			0.994^{ns}
≤3	15 (2.19%)	15 (2.18%)	
>3	670 (97.81%)	672 (97.82%)	
Nut Consumption Frequency (times/week)			0.959^{ns}
≤3	631 (92.79%)	637 (92.72%)	
>3	49 (7.21%)	50 (7.28%)	
Smoking			1.000^{ns}
Current smoker	15 (2.20%)	15 (2.18%)	
Former smoker	5 (0.73%)	5 (0.73%)	
Never smoked	661 (97.06%)	667 (97.09%)	
Macrosomia			0.992^{ns}
Yes	61 (9.15%)	62 (9.02%)	
No	585 (87.71%)	604 (87.92%)	
Uncertain	21 (3.15%)	21 (3.06%)	
PIH			0.974^{ns}
Yes	21 (3.15%)	21 (3.06%)	
No	524 (78.68%)	544 (79.18%)	
Uncertain	121 (18.17%)	122 (17.76%)	

Continued Supplementary Table 1 Comparison of Data Distribution Between Original Data and Imputed Data

Variables	Original Data	Imputed Data	P
Gestational Diabetes Mellitus			0.991[ns]
Yes	2 (0.30%)	2 (0.29%)	
No	541 (81.23%)	560 (81.51%)	
Uncertain	123 (18.47%)	125 (18.20%)	

ns: The difference was not statistically significant; SBP, Systolic blood pressure; DBP, Diastolic blood pressure; BMI, Body mass index; PIH, Pregnancy-induced hypertension; E2, Estradiol; TG, Triglycerides; LDL, Low-density lipoprotein; HDL, High-density lipoprotein; AST, Aspartate aminotransferase; TP, Total protein; ALB, Albumin; GLB, Globulins; A/G, Albumin/Globulin ratio; PT, Prothrombin time; UA, Uric acid; Scr, Serum creatinine; GFR, Glomerular filtration rate; CysC, Cystatin C.

Supplementary Table 2 The Difference Between the Training Set and Internal Validation Set

**Parameter**	**Training Set (n=481)**	**Internal Validation Set (n=206)**	**P**
Patient Distribution			0.904^{ns}
Control	384 (79.83%)	166 (80.58%)	
Case	97 (20.17%)	40 (19.42%)	
Age (years)	53.00 (50.00, 56.00)	54.00 (49.00, 56.00)	0.881^{ns}
Menarche Age (years)	14.00 (13.00, 14.00)	14.00 (13.00, 14.00)	0.193^{ns}
Age at First Birth (years)	24.00 (22.00, 25.00)	24.00 (22.00, 25.00)	0.581^{ns}
Sleep Duration (hours)	7.00 (5.00, 8.00)	7.00 (6.00, 8.00)	0.306^{ns}
BMI (kg/m^2)	23.31 (21.50, 25.48)	23.10 (21.36, 25.33)	0.590^{ns}
Heart Rate (bpm)	76.00 (68.00, 84.00)	76.50 (67.75, 84.00)	0.817^{ns}
Abdominal Circumference (cm)	83.00 (78.00, 88.00)	83.00 (78.00, 89.00)	0.506^{ns}
SBP (mmHg)	133.00 (120.00, 146.50)	134.00 (120.00, 146.75)	0.771^{ns}
DBP (mmHg)	80.00 (70.00, 90.00)	80.00 (71.00, 87.00)	0.602^{ns}
E2 (pg/mL)	22.10 (14.60, 31.55)	21.80 (14.75, 30.18)	0.633^{ns}
Testosterone (nmol/mL)	0.36 (0.26, 0.42)	0.36 (0.28, 0.43)	0.815^{ns}
TG (mmol/L)	1.55 (1.13, 2.17)	1.47 (1.05, 2.05)	0.220^{ns}
Total Cholesterol (mmol/L)	4.54 (3.89, 5.22)	4.42 (3.78, 5.14)	0.273^{ns}
LDL (mmol/L)	2.65 (2.16, 3.28)	2.57 (2.02, 3.23)	0.147^{ns}
HDL (mmol/L)	1.22 (1.03, 1.43)	1.21 (1.04, 1.41)	0.793^{ns}
Alanine Transaminase (U/L)	17.70 (12.55, 26.00)	16.50 (12.50, 24.28)	0.546^{ns}
AST (U/L)	20.07 (17.00, 24.57)	20.05 (17.06, 24.55)	0.840^{ns}
TP (g/L)	65.40 (61.10, 69.30)	64.45 (60.90, 68.33)	0.119^{ns}
ALB (g/L)	41.00 (38.40, 43.55)	40.50 (38.62, 43.10)	0.441^{ns}
GLB (g/L)	24.10 (21.55, 26.70)	23.40 (21.50, 27.03)	0.501^{ns}
A/G (Ratio)	1.70 (1.52, 1.92)	1.70 (1.51, 1.93)	0.870^{ns}

Continued Supplementary Table 2 The Difference Between Training Set and Internal Validation Set

Parameter	Training Set (n=481)	Internal Validation Set (n=206)	P
PT (s)	9.30 (2.75, 11.10)	9.20 (2.80, 11.10)	0.558^{ns}
UA (μmol/L)	250.00 (6.25, 336.55)	256.10 (6.00, 335.93)	0.605^{ns}
Scr (μmol/L)	56.00 (48.81, 65.97)	54.60 (48.59, 65.82)	0.672^{ns}
Blood Urea Nitrogen (mmol/L)	5.72 (4.45, 231.75)	5.59 (4.02, 223.90)	0.313^{ns}
GFR (mL/min/1.73m²)	72.57 (25.10, 97.73)	68.40 (24.88, 98.28)	0.600^{ns}
CysC (mg/L)	0.88 (0.67, 1.10)	0.83 (0.62, 1.03)	0.051^{ns}
Residential Area			0.708^{ns}
Urban	229 (47.61%)	102 (49.51%)	
Rural	252 (52.39%)	104 (50.49%)	
Menopause			0.925^{ns}
Yes	368 (76.51%)	159 (77.18%)	
No	113 (23.49%)	47 (22.82%)	
Use of Health Supplements			0.689^{ns}
Yes	81 (16.84%)	38 (18.45%)	
No	400 (83.16%)	168 (81.55%)	
Alcohol Consumption			0.565^{ns}
Yes	11 (2.29%)	7 (3.40%)	
No	470 (97.71%)	199 (96.60%)	
Coffee Consumption			1.000^{ns}
Yes	8 (1.67%)	3 (1.46%)	
No	473 (98.34%)	203 (98.54%)	
Tea Consumption			0.350^{ns}
Yes	93 (19.33%)	47 (22.82%)	
No	388 (80.67%)	159 (77.18%)	
Hypertension			0.384^{ns}
Yes	208 (43.24%)	81 (39.32%)	
No	273 (56.76%)	125 (60.68%)	

Continued Supplementary Table 2 The Difference Between Training Set and Internal Validation Set

Parameter	Training Set (n=481)	Internal Validation Set (n=206)	P
Education Level			0.446^{ns}
Illiterate	3 (0.62%)	1 (0.49%)	
Primary School	75 (15.60%)	32 (15.53%)	
Junior High School	212 (44.07%)	93 (45.15%)	
High School or Technical Secondary School	123 (25.57%)	61 (29.61%)	
College or Higher	68 (14.14%)	19 (9.22%)	
Physical Activity			0.531^{ns}
Almost no exercise	182 (37.84%)	86 (41.75%)	
Irregular exercise	170 (35.34%)	72 (34.95%)	
Regular exercise	129 (26.82%)	48 (23.30%)	
Sedentary Time (hours)			0.720^{ns}
1-3	77 (16.00%)	33 (16.02%)	
3-5	264 (54.89%)	113 (54.85%)	
5-7	115 (23.91%)	45 (21.84%)	
>7	25 (5.20%)	15 (7.29%)	
Meat Consumption Frequency			0.057^{ns}
Never	28 (5.82%)	4 (1.94%)	
Occasionally	302 (62.79%)	142 (68.93%)	
Often	151 (31.39%)	60 (29.13%)	
Types of Meat Consumed			0.487^{ns}
Pork	393 (81.70%)	178 (86.41%)	
Chicken, Duck	41 (8.52%)	14 (6.80%)	
Beef, Lamb	5 (1.04%)	0 (0.00%)	
Fish	19 (3.95%)	5 (2.43%)	
Other	23 (4.78%)	9 (4.37%)	
Egg Consumption Frequency (times/week)			0.703^{ns}
≤3	341 (70.90%)	149 (72.30%)	
>3	140 (29.10%)	57 (27.60%)	

Continued Supplementary Table 2 The Difference Between Training Set and Internal Validation Set

**Parameter**	**Training Set (n=481)**	**Internal Validation Set (n=206)**	**P**
Soy Product Consumption Frequency (times/week)			0.385[ns]
≤3	446 (92.72%)	187 (90.77%)	
>3	35 (7.28%)	19 (9.23%)	
Dairy Product Consumption Frequency (times/week)			0.190[ns]
≤3	426 (88.56%)	175 (84.95%)	
>3	55 (11.43%)	31 (15.05%)	
Fruit Consumption Frequency (times/week)			0.518[ns]
≤3	279 (58.00%)	114 (55.34%)	
>3	202 (42.00%)	92 (44.66%)	
Vegetable Consumption Frequency (times/week)			0.777[ns]
≤3	11 (2.29%)	4 (1.95%)	
>3	470 (97.72%)	202 (98.06%)	
Nut Consumption Frequency (times/week)			0.210[ns]
≤3	442 (91.89%)	195 (94.66%)	
>3	39 (8.11%)	11 (5.34%)	
Smoking			0.462[ns]
Current smoker	10 (2.08%)	5 (2.43%)	
Former smoker	5 (1.04%)	0 (0.00%)	
Never smoked	466 (96.88%)	201 (97.57%)	
Macrosomia			0.703[ns]
Yes	43 (8.94%)	19 (9.22%)	
No	425 (88.36%)	179 (86.89%)	
Uncertain	13 (2.70%)	8 (3.88%)	
PIH			0.722[ns]
Yes	15 (3.12%)	6 (2.91%)	
No	377 (78.38%)	167 (81.07%)	
Uncertain	89 (18.50%)	33 (16.02%)	

Continued Supplementary Table 2 The Difference Between Training Set and Internal Validation Set

**Parameter**	**Training Set (n=481)**	**Internal Validation Set (n=206)**	**P**
Gestational Diabetes Mellitus			0.836[ns]
Yes	2 (0.42%)	0 (0.00%)	
No	389 (80.87%)	171 (83.00%)	
Uncertain	90 (18.71%)	35 (16.99%)	

ns: The difference was not statistically significant; SBP, Systolic blood pressure; DBP, Diastolic blood pressure; BMI, Body mass index; PIH, Pregnancy-induced hypertension; E2, Estradiol; TG, Triglycerides; LDL, Low-density lipoprotein; HDL, High-density lipoprotein; AST, Aspartate aminotransferase; TP, Total protein; ALB, Albumin; GLB, Globulins; A/G, Albumin/Globulin ratio; PT, Prothrombin time; UA, Uric acid; Scr, Serum creatinine; GFR, Glomerular filtration rate; CysC, Cystatin C.

Supplementary Table 3 Lasso-Selected Variable Coefficients and VIFs

Variables	Intercept	VIF
Education level	-0.057	1.119
Menarche Age	0.040	1.023
Egg consumption frequency	0.411	1.104
Nut consumption frequency	-0.015	1.055
Coffee Consumption	0.506	1.086
PIH	-0.002	5.655
Gestational Diabetes Mellitus	-0.297	5.702
Heart Rate	0.000	1.029
Testosterone	0.039	1.020
LDL	0.129	1.163
HDL	-0.047	1.177
AST	0.010	1.008
TP	0.044	1.056
PT	0.019	1.042
GFR	-0.033	1.065
CysC	0.592	1.091

PIH, Pregnancy-induced hypertension; LDL, Low-density lipoprotein; HDL, High-density lipoprotein; AST, Aspartate aminotransferase; TP, Total protein; PT, Prothrombin time; GFR, Glomerular filtration rate; CysC, Cystatin C.

Supplementary Table 4 Binary Logistic Regression with Variables Added: Age, Estradiol

Variables	B	P	OR	95% CI lower limit	95% CI upper limit
Education Level (ref: College or Higher)		0.244			
Illiterate		0.101	13.546	0.602	304.809
Primary School		0.961	0.975	0.352	2.699
Junior High School		0.927	0.961	0.404	2.284
High School or Technical Secondary School		0.276	0.587	0.225	1.531
Menarche Age	0.182	0.078	1.200	0.979	1.471
Egg Consumption Frequency (ref: More than three time)	-0.603	0.047	0.547	0.301	0.993
Nut Consumption Frequency (ref: More than three time)	0.698	0.244	2.010	0.621	6.513
Coffee Consumption (ref: Yes)	-20.417	0.999	0.000	0.000	.
PIH (ref: Uncertain)		0.844			
Yes		1.000	0.000	0.000	.
No		1.000	0.000	0.000	.
Gestational Diabetes Mellitus (ref: Uncertain)		1.000			
Yes		1.000	6.211	0.000	.
No		1.000	505820841.182	0.000	.
Heart Rate	-0.011	0.152	0.989	0.974	1.004
Testosterone	1.671	0.093	5.316	0.758	37.290
LDL	0.350	0.040	1.419	1.016	1.981
HDL	-0.799	0.102	0.450	0.173	1.172
AST	0.011	0.124	1.011	0.997	1.025
TP	0.068	0.002	1.070	1.025	1.117
PT	0.040	0.226	1.041	0.975	1.112
GFR	0.000	0.991	1.000	0.993	1.007
CysC	2.222	<0.001	9.230	4.500	18.932
Age	0.015	0.680	1.015	0.945	1.091
E2	0.005	0.617	1.005	0.987	1.023

PIH, Pregnancy-induced hypertension; E2, Estradiol; LDL, Low-density lipoprotein; HDL, High-density lipoprotein; AST, Aspartate aminotransferase; TP, Total protein; PT, Prothrombin time; GFR, Glomerular filtration rate; CysC, Cystatin C.

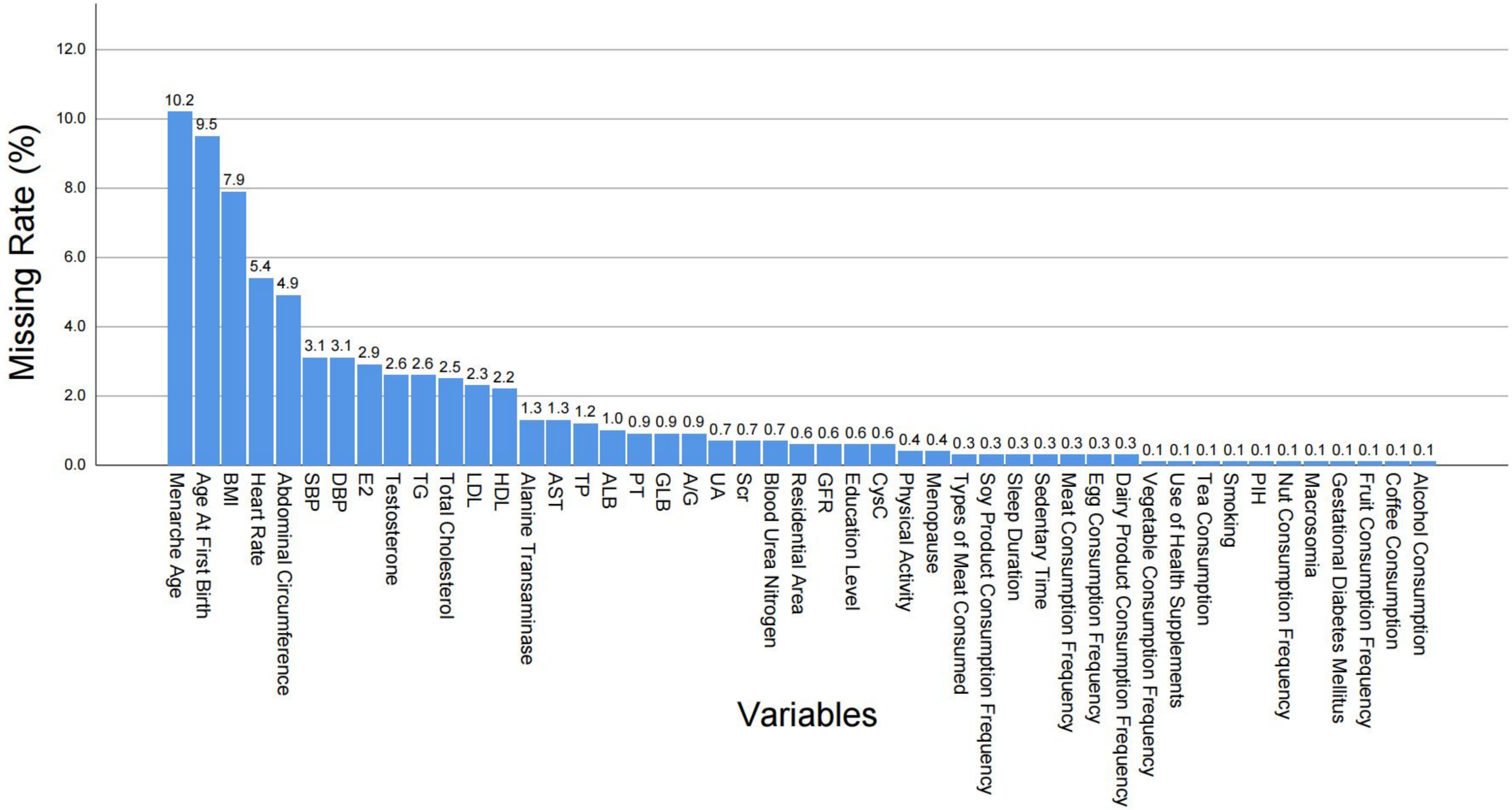

Supplementary Figure 1 Distribution of Missing Rates Across Variables

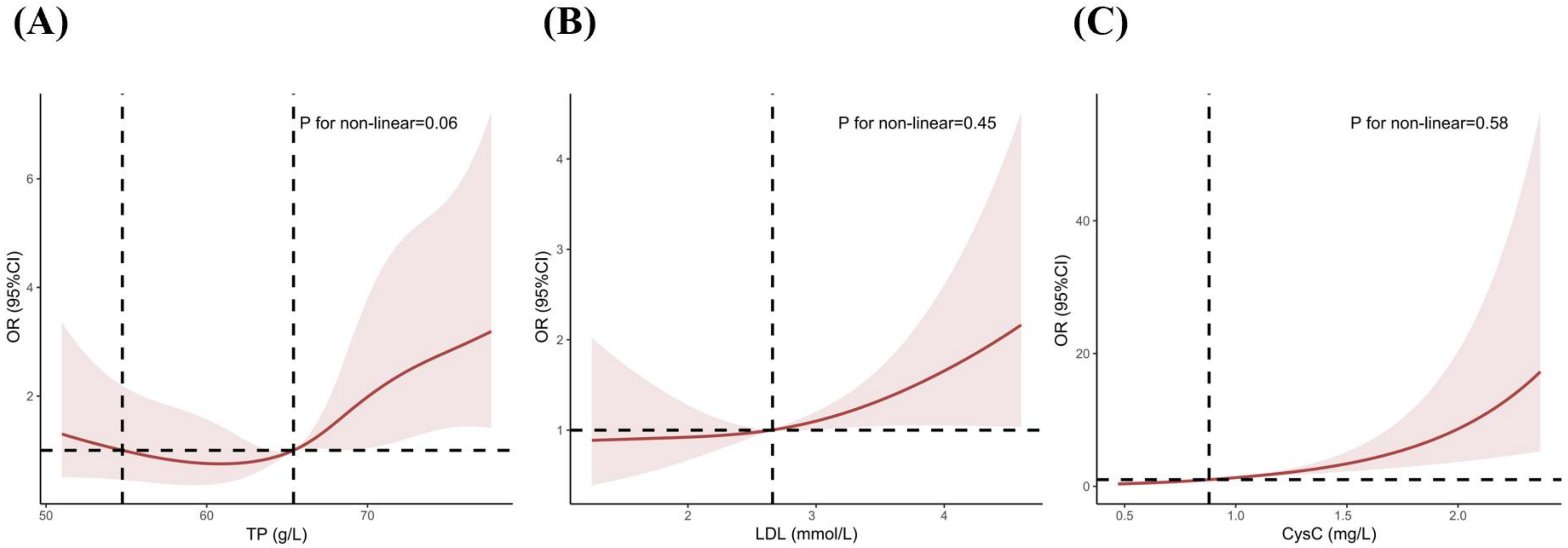

Supplementary Figure 2 Restricted Cubic Spline (RCS) Analysis of the Relationship Between Continuous Variables and HHcy Prevalence

(A) RCS plot illustrating the linear relationship between TP and HHcy prevalence; (B) RCS plot showing the linear relationship between LDL and HHcy prevalence; (C) RCS plot depicting the linear relationship between CysC and HHcy prevalence.
